# *Mycoplasma genitalium*: whole genome sequence analysis, recombination and population structure

**DOI:** 10.1186/s12864-017-4399-6

**Published:** 2017-12-28

**Authors:** Maria C. Fookes, James Hadfield, Simon Harris, Surendra Parmar, Magnus Unemo, Jørgen S. Jensen, Nicholas R. Thomson

**Affiliations:** 1 Pathogen Genomics, The Wellcome Trust Sanger Institute, Wellcome Trust Genome Campus, Hinxton, Cambridge, UK; 2 Clinical Microbiology and Public Health Laboratory, National Infection Service, Cambridge University Hospitals NHS Foundation Trust, Hills Road, Cambridge, UK; 3 WHO Collaborating Centre for Gonorrhoea and other Sexually Transmitted Infections, Department of Laboratory Medicine, Microbiology, Faculty of Medicine and Health, Örebro University, Örebro, Sweden; 4 Division for Infection Preparedness, Bacteria, Parasites, and Fungi, Research Unit for Reproductive Tract Microbiology, Statens Serum Institute, Copenhagen, Denmark; 5 London School of Hygiene and Tropical Medicine, London, UK

**Keywords:** *Mycoplasma genitalium* genomics and phylogenetics, STIs, Azithromycin resistance, *Mycoplasma genitalium*

## Abstract

**Background:**

Although *Mycoplasma genitalium* is a common sexually transmitted pathogen causing clinically distinct diseases both in male and females, few genomes have been sequenced up to now, due mainly to its fastidious nature and slow growth. Hence, we lack a robust phylogenetic framework to provide insights into the population structure of the species. Currently our understanding of the nature and diversity of *M. genitalium* relies on molecular tests targeting specific genes or regions of the genome and knowledge is limited by a general under-testing internationally. This is set against a background of drug resistance whereby *M. genitalium* has developed resistance to mainly all therapeutic antimicrobials.

**Results:**

We sequenced 28 genomes of *Mycoplasma genitalium* from temporally (1980–2010) and geographically (Europe, Japan, Australia) diverse sources. All the strain showed essentially the same genomic content without any accessory regions found. However, we identified extensive recombination across their genomes with a total of 25 regions showing heightened levels of SNP density. These regions include the MgPar loci, associated with host interactions, as well as other genes that could also be involved in this role. Using these data, we generated a robust phylogeny which shows that there are two main clades with differing degrees of genomic variability. SNPs found in region V of 23S rRNA and *parC* were consistent with azithromycin/erythromycin and fluoroquinolone resistances, respectively, and with their phenotypic MIC data.

**Conclusions:**

The sequence data here generated is essential for designing rational approaches to type and track *Mycoplasma genitalium* as antibiotic resistance increases. It represents a first approach to its population genetics to better appreciate the role of this organism as a sexually transmitted pathogen.

**Electronic supplementary material:**

The online version of this article (10.1186/s12864-017-4399-6) contains supplementary material, which is available to authorized users.

## Background

*Mycoplasma genitalium* is a pathogenic member of the *Mycoplasmataceae* restricted to humans, primarily colonizing the urogenital tract, and causing sexually transmitted infections in both males and female [1–3]. It is closely related to the important respiratory tract pathogen *M. pneumoniae* which has a larger genome size of 816 kbp [4] compared to the smaller genome of only 580 kbp for the *M. genitalium* reference strain G37^T^ [5]*.* However, all of the 470 proposed *M. genitalium* coding sequences have orthologues in *M. pneumoniae* [6]. There are many other human-associated *Mycoplasma* species, including *M. hominis*, *Ureaplasma urealyticum* and *U. parvum*. However, the clinical importance of these species is disputed, particularly when they are confined to the lower urogenital tract [7].

*M. genitalium* causes urethritis in males, and urethritis and cervicitis in females [2]. If not detected and appropriately treated, these infections may result in pelvic inflammatory disease (PID), preterm birth, spontaneous miscarriage, and tubal factor infertility [8]. NAATs are the only feasible, sensitive and specific diagnostic methods for *M. genitalium* infections, but access to testing for *M. genitalium* is limited in many settings [9]. Consequently, epidemiological data regarding *M. genitalium* infections are relatively scarce internationally. This is of concern because *M. genitalium* can develop resistance to nearly all current antimicrobials introduced for treatment and a range of novel antimicrobials, therefore molecular resistance testing is crucial [10, 11].

The archetypal *M. genitalium* isolates, strains G37^T^ and M30*,* were identified in 1980 from two male patients with NGU in the UK [12]. The isolates were recovered from SP4 mycoplasma medium after >50 day’s incubation and were shown to represent a new species [12]. *M. genitalium* G37^T^ was selected as the type strain and in 1995, was the first *Mycoplasma* to be whole genome sequenced [5] showing that it had, at the time, the smallest known genome (0.58 Mb) of any free-living organism, predicted to encode fewer than 500 proteins. As a consequence, *M. genitalium* was shown to have a reduced metabolic capacity, lacking the enzymes for amino- and fatty acid biosynthesis [5]. This reduced genome, like that of other host- or niche-restricted pathogens, is thought to be the result of a genomic streamlining, where genes that encode functions important for its former lifestyle are lost as the microorganism adapts to a specific host and niche within that host [13, 14]. Other characteristics of the *M. genitalium* genome are the general lack of regulatory systems, the low number of transport systems and, most remarkably, the presence of an expanded family of repetitive chromosomal repeats, known as MgPar regions [5]. The 9 MgPar loci within *M. genitalium* G37^T^ accounted for over 4% of the genome and have been shown to be involved in antigenic variation [15, 16]. The MgPar loci represent non-functional fragments of specific genes, *mgpBC,* which are functional and located within a single expression site on the chromosome [5], flanked by MgPar4 upstream and MgPar5 downstream. The genes *mgpBC* contain trinucleotide tandem repeat regions and have been shown to encode a cytoadhesin (P140) and a conserved hypothetical protein (P110), respectively, both of which are involved in cytoadherence [17]. The MgPar regions represent a reservoir of variation that can be rapidly introduced into the *mgpBC* gene sequence by recombination between the genes in the expression site and the various MgPar loci [18]. MgpB is a major surface antigen of *M. genitalium* and so this is proposed to increase antigenic diversity as a mechanism of immune evasion, which in turn has been linked to persistence in the human reproductive tract [16, 19]. In addition, due to the inherent variability of the *mgpBC* genes they have been used as the basis of most of the established typing schemes for *M. genitalium* [20, 21].

Despite their importance to human health, there are only 5 fully sequenced *M. genitalium* genomes to date, largely because of the fastidious nature and slow growth of the organism. They include the recent draft genomes of four *M. genitalium* strains isolated from patients in Denmark, Japan, and Australia [22], which were obtained from axenic cultures raised from single colonies and took up to a year to grow from a swab or urine specimens; a procedure normally involving 2 to 15 passages in Vero cells [23, 24]. To increase our understanding of the population framework and diversity of *M. genitalium* we sequenced a set of 21 geographically and temporally diverse *M. genitalium* strains (1 of them with three isolates from the same patient) as well as other 5 strains available from the ATCC. In doing so we present a phylogenetic snapshot of the species by sequencing a collection of isolates originating from three continents between 1980 and 2010.

## Results

### *Mycoplasma genitalium* isolates, sequencing and variation

A total of 28 *M. genitalium* genomes were sequenced. These included 22 isolates obtained by the Vero cell culture method from 20 patients between 1991 and 2010. Three of these isolates originated from a single French patient collected over a period of 79 days (M6151 day 0; M6090 day 41, and M6312 day 79). Two strains were from samples from Japanese patients collected in 2003 and three were from Australian patients collected in 2004 and 2010 (summarised in Table 1). In addition, an early passage *M. genitalium* M30 strain was also included that originated from samples collected in the UK in 1980, and finally five additional ATCC reference strains were also sequenced: R32G, TW10-6G, TW10-5G, TW48–5G [25] and UTMB-10G [26], described as being isolated from extra-genital samples.

**Table 1:** Main characteristics of the *Mycoplasma genitalium* sample set (*n* = 28)

Sample Name	Country of Origin	Year of Isolation	AZM^b^	ERY^b^	CIP^b^	MXF^b^	DOX^b^	SOL^b^
M2282	Denmark	1991	0.004	0.063	4	0.125	0.25	<= 0.001
M2300	Denmark	1991	0.008	0.125	16	0.125	0.25	<= 0.001
M2341	Denmark	1991	0.008	0.25	2	0.25	1	<= 0.001
M30	UK	1980	0.008	0.125	8	0.125	0.5	<= 0.001
M6090^a^	France	1994	0.004	0.063	2	0.063	0.125	<= 0.001
M6151^a^	France	1994	0.008	0.125	4	0.125	0.25	<= 0.001
M6257	Sweden	2004	>8	>16	1	0.25	0.5	2
M6270	Australia	2004	>8	>16	4	0.125	0.25	0.5
M6280	Sweden	1997	0.004	0.063	1	0.063	0.125	<= 0.001
M6283	Japan	2003	0.004	0.063	2	0.125	0.5	<= 0.001
M6284	Japan	2003	0.004	0.125	4	0.125	0.25	<= 0.001
M6285	Sweden	1997	0.004	0.125	1	0.125	0.5	<= 0.001
M6286	Sweden	2001	0.002	0.03	2	0.03	0.25	<= 0.001
M6303	Norway	2003	>8	>16	8	0.25	2	0.5
M6312^a^	France	1994	0.004	0.063	8	0.125	0.25	<= 0.001
M6327	Denmark	2005	0.016	0.125	8	0.25	0.25	0.002
M6328	Sweden	1998	0.004	0.125	2	0.125	0.25	<= 0.001
M6475	Sweden	2006	0.008	0.125	1	0.5	0.5	<= 0.001
M6489	Sweden	2007	>16	>16	>16	>16	1	1
M6593	Norway	2008	16	>16	2	0.125	0.063	0.5
M6604	Denmark	2009	64	>16	4	0.25	0.5	2
M6711	Australia	2010	>16	> = 64	>16	8	1	0.25
M6713	Australia	2010	0.063	0.03	0.5	0.125	0.5	<= 0.001
R32G	USA	1974-1975^c^	ND	ND	ND	ND	ND	ND
TW10-6G	USA	1974-1975^c^	ND	ND	ND	ND	ND	ND
TW10-5G	USA	1974-1975^c^	ND	ND	ND	ND	ND	ND
TW48–5G	USA	1974-1975^c^	ND	ND	ND	ND	ND	ND
UTMB-10G	USA	1986^d^	ND	ND	ND	ND	ND	ND

*ND*: Not determined

^a^Samples isolated from the same patient

^b^MIC values in μg/ml

^c^Baseman et al. [25]

^d^Tully et al. [26]

De novo assemblies were generated from the Illumina read data for each isolate (see methods). Twelve of the 28 genomes assembled into a single contig (without scaffolding), with the most fragmented genome comprising only 6 contigs (Additional file 1: Table S1). All genomes were syntenic with respect to the published reference G37^T^ [5] and ranged in size from 579,938 to 586,920 bp, with the most extreme differences seen for isolates M6327, which was 138 bp shorter and M6713, which was 6844 bp longer than the G37^T^ reference genome (see Table 1 and Additional file 1: Table S1). The additional 6.8 kb of sequence carried by *M. genitalium* strain M6713 was entirely explained by the expansion of the number of repeats within a single MgPar locus (see below). In total 8 other isolates exceeded the reference’s genome length by 1 kb or more (see Additional file 1: Table S1) this was all explained by expansion of the number of MgPar regions and/or their repeats or a small number of single base insertions.

Assembled whole genome data showed that there was less than 0.5% nucleotide divergence, in pairwise comparisons, between any genome sequenced in this study when compared to the G37^T^ reference sequence (range was 0.039% for strain M6151 to 0.424% for the M6283; Additional file 1: Table S1). This analysis also showed that all isolates had a similar GC content (~31.7%) and the number of predicted coding sequences (CDSs) was 482 for all isolates, identical to the reference G37^T^ genome [5, 27].

For completeness we also sequenced the five additional ATCC reference strains: R32G, TW10-6G, TW10-5G, TW48–5G [25] and UTMB-10G [26], described as being isolated from extra-genital samples. The genomes of the five ATCC reference strains showed only minor variation compared to *M. genitalium* G37^T^, amounting to no whole gene differences and at a maximum of 2–14 SNPs. However, if recombination regions were ignored (using Gubbins; see below for a detailed description of the contribution of recombination to genome variation) then 3 of the 5 strains were in fact identical to G37^T^ and 2 samples (UTMP and TW48–5) differed by only a single base pair from the reference strain G37^T^. Of note the variation was the same (C - > A) and at the same site in both isolates (reference base position 340,583), which represented a synonymous change on the gene MG_279). As these strains are almost identical to the reference strain G37^T^, our subsequent analyses are based on the remaining 23 genomes sequenced on this work with the addition of the publicly available genomes.

### The *Mycoplasma genitalium* phylogeny

To construct a whole genome SNP-based phylogeny, sequence reads were mapped to the published genome of the *M. genitalium* G37^T^ [5] (see methods; Additional file 2: Table S2). The minimum percentage of the G37^T^ genome covered by reads for any of the isolates sequenced here was 98.49% (isolate M6283). The average depth of mapping coverage for all of the samples was 230× (Additional file 1: Table S1). From the mapped read data, the number of SNPs identified in each sample with respect to the reference ranged from 225 to 2473 for samples M6151 and M6283, respectively, with a total of 11,028 variable sites across the genome. The total number of SNPs per isolate compared to G37^T^ is summarised in Additional file 1: Table S1 (including homoplasic SNPs, see below).

**Fig. 1:**
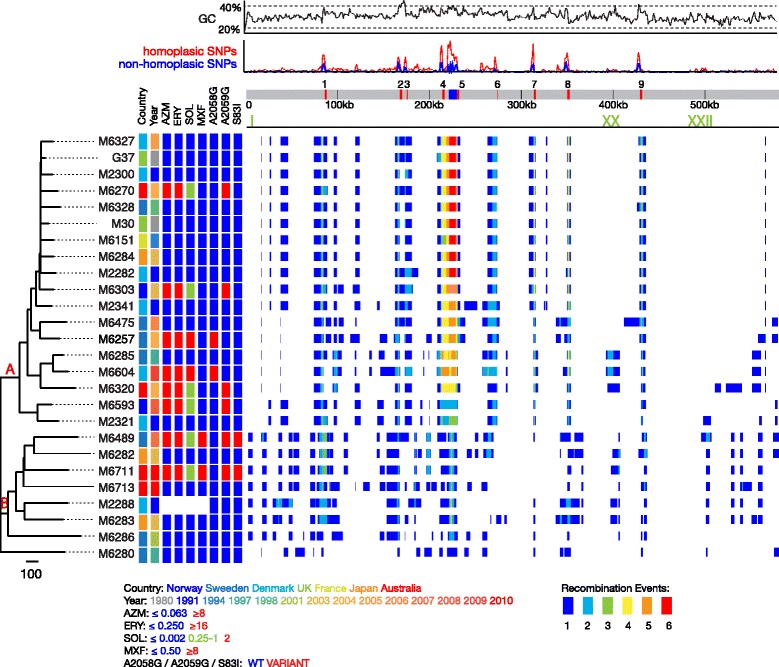
Phylogenetic tree of *Mycoplasma genitalium.* Phylogenetic tree showing the nodes separating the two main clades A and B (in red). MICs in μg/ml. A2058G and A2059G refers to nucleotide positions of the 23S rRNA sequence while S83I is the amino acid change on *parC*. Grey bar represents the *M. genitalium* G37^T^ genome along which coloured blocks representing: red, MgPar loci (numbered 1–9); blue, the *mgp* operon. Below, specific recombination regions I, XX and XXII are indicated in green. Main frame represents a heatmap showing the number of independent recombinations detected at that site. Above, graphs showing the relative number of SNPs (split into Non-homoplasic and homoplasic as labelled) across the *M. genitalium* G37^T^ genome, calculated using Gubbins and the genome GC content are plotted

*M. genitalium,* like other *Mycoplasma* species, has been shown to exhibit high rates of recombination [28]. We used compatibility-based recombination detection methods to assess recombination in the *M. genitalium* genomes. All methods showed unequivocally strong signals (*p*-values <0.01) of recombination across all *M. genitalium* genomes sequenced in this study (see methods, data not shown). In order to estimate an accurate phylogeny, we sought to mask out regions of recombination. In total ~60% of the 11,028 total variable sites identified across all isolates were predicted to be homoplasic by Gubbins (see methods; Fig. 1). Once these SNPs had been masked out, we constructed a maximum likelihood (ML) whole genome phylogeny based on the remaining total number of 4196 variable sites across the 26 isolate genomes (Fig. 1). Comparing phylogenies constructed with and without recombination removed, it is clear that whilst overall topology of the tree remains the same, the branch lengths were shorter in the phylogeny with recombination removed, as would be expected (Additional file 3: Figure S1).

The phylogenetic tree in Fig. 1 shows that the *M. genitalium* isolates form two main clades comprised of 18 (Clade A) and 7 (Clade B) isolates, respectively. The existence of the two clades is well supported (see Additional file 4: Figure S2 for bootstrap values). We used the software package Bayesian Analysis of Population Structure (BAPS) to identify robust Phylogenetic Groups (PGs) within *M. genitalium.* BAPS identified two PGs, (probability 1, marginal likelihood of the optimal partition −37,393.6227 and a reduced likelihood for any change of group ranging from −267.1 and −1093.5), in agreement with the simple population structure designation except for sample M6280, which was reassigned to Clade B. Unlike many other bacterial genomes and considering all SNPs across the phylogeny, there was no obvious correlation between the root to tip phylogenetic distances and the year of isolation (Additional file 5: Figure S3). Moreover, we also found no clustering either to the country of origin or the year of isolation (Fig. 1).

### Genes falling within recombination blocks

Using Gubbins to identify regions of elevated SNP density (a marker of recombination) it was possible to identify 339 recombination blocks representing independent recombination events across the genomes, with distinct borders. These blocks often overlapped to reveal 25 recombination regions composed of contiguous or overlapping recombination blocks, ranging in size from 1035 to 46,265 bp (Fig. 1; Additional file 2: Table S2).

Looking across all isolates only one recombination block, encompassing 7 genes ranging from *gyrA* to MG_009, was uniquely associated to any particular clade (Clade B; coordinates 6855..11884; region I in Additional file 2: Table S2; Fig. 1). Some of the regions of recombination appeared as ‘hotspots’ for recombination because there is evidence of recombination having occurred multiple times at that site (Fig. 1). Based on all isolates mapped to the reference, this data show that as much as 66% of the genome of G37^T^ falls under a recombination block from one or more of our sequenced set of samples. On average, 50% of each isolate’s genome is predicted to be recombinant. In total, 375 CDSs were encoded within recombination blocks. The most frequent recombination events were centred around the *M. genitalium* MgPar4 and MgPar5 loci (Fig. 1), although all of the MgPar loci were hotspots, with the recombination blocks generally extending both upstream and downstream of these loci. Recombination between the MgPar loci has been noted previously on a limited number of isolates and shown to include both reciprocal and non-reciprocal intramolecular recombination events [15, 18, 29]. However, it is evident from Fig. 1 that the MgPar regions are not the only regions of recombination across the genome. Other recombination blocks were found to span genes *hmw*2 (257,662..278755; region XIII) and *hmw*1 and *hmw*3 (391,075..406322; region XX Additional file 2: Table S2) genes which in *M. pneumoniae* encode proteins involved in cytoadherence [5, 30].

Finally, a region between genes MG_390 and *atp*D (493,411..504572; region XXII, Additional file 2: Table S2), encompassing MG_395 and MG_397, which is predicted to encode a lipoprotein and a hypothetical protein, respectively, were also found within recombination regions; gene MG_395 shares, 64–74% nucleotide similarity, to the lipoprotein-encoding genes, MG_067 and MG_068, respectively, which are located immediately upstream of MgPar1 and are included in the recombination block of this first MgPar region. None of these listed loci contained recognised short sequence repeats similar to the ones found within the *mgpBC* genes.

### Recombination between *MgPar* loci and the *mgpABC* expression site

The MgPar regions are composed of 3 discrete repeat units that match regions in *mgpB,* designated B, EF and G, and a composite repeat unit that matches region(s) in *mgpC*. The *mgpC* repeat is designated JKLM [15, 17, 18, 29, 31]; see Additional file 6: Figure S4). In all fully sequenced *M. genitalium* genomes and across our collection where the contiguation is unambiguous, the JKLM repeat can be found in MgPar loci as a complete unit, as is found in the *mgpC* gene locus itself, as a truncated sequence (as seen in MgPar locus1) or split (JKLM-1 and JKLM-2) and truncated (as found in MgPar loci 3, 4, 5 and 7). It is clear that not all MgPar regions contain all of the repeated sequences [this study; [15, 18, 31]: The G repeat is always found after the shorter JKLM or inserted between the 2 split JKLM fragments: JKLM-1 and JKLM-2 (see Additional file 6: Figure S4).

To study the dynamics of recombination in the MgPar loci and *mgpBC* expression site genes, we postulated that we could use the disruption of phylogenetic signal for each of the multiple homologous repeat sequences within the MgPar loci and the *mgpC* gene itself to determine the nature, extent and rapidity of recombination in these regions. To do this, we first analysed the genomes of isolates M6151, M6090 and M6312 which were collected sequentially from the same French patient over 79 days (Table 1). The whole genome data from these three isolates showed that only 7 SNPs differentiate M6312 from M6090 or M6151 (after recombination was removed). We reconstructed the phylogeny of each of the B, EF and G repeat sequences. The JKLM repeat was split so that phylogenies for JKLM-1 and JKLM-2 could be constructed independently.

Within the timeframe of the sampling of this patient we could see disruption of the phylogenies for the EF, JKLM-1 and JKLM-2 repeat sequences in different MgPar loci (Additional file 7: Figure S5). The SNP profile of the different EF repeats alongside the isolates EF repeats phylogeny (Additional file 7: Figure S5, for EF repeat, right), show in detail examples of both homologous and non-reciprocal recombination, with exchange of whole repeats and partial repeats, consistent with previous reports [5, 15, 18, 31]. For example, for the *mgpB* location*,* the EF repeat of isolates M6090 and M6151 are identical while in sample M6312 the repeat at the same location has sequence signatures of both the EF repeats from MgPar2 and MgPar6. This is reflected in the specific phylogeny for these 3 genomic locations (Additional file 6: Figure S4, for EF, tree on the right).

Perhaps unsurprisingly considering the complex recombination events seen in a single patient when we extended this analysis to all of the MgPar/*mgpBC* repeats found in all samples and locations all phylogenetic signal was lost, with no clustering by genomic location or sample or whole genome phylogenetic position, (Additional file 8: Figure S6). However, these data did imply that both intra- and inter-specific recombination occurred in isolates of the same and different clades of the tree. The only exception to this was for repeats G and JKLM-2 which in many samples showed an identical sequence at the same location (MgPar loci 3, 5 and 7). It is notable that these represent 3 of the 4 MgPar loci where the repeat G is inserted into the JKLM repeat splitting it into in the two segments [15, 17, 18, 32].

### Antibiotic resistance of *Mycoplasma genitalium* isolates

Antibiotic resistance phenotypic profiles (measured as MICs (μg/ml)) for 6 antibiotics relevant (present or past) for the treatment of *M. genitalium* infections and other sexually transmitted pathogens, were determined for all isolates (see Table 1). Isolates comprised seven azithromycin resistant strains (MIC > 8 μg/ml), two of which were multidrug resistant with accompanying moxifloxacin resistance (MIC ≥ 8 μg/ml). Erythromycin and ciprofloxacin showed a much lower activity against the *M. genitalium* isolates than azithromycin and moxifloxacin. Doxycycline MICs would generally be considered well within the in vitro susceptible range (MIC ≤ 2 μg/ml), although one strain (M6303) had an MIC of 2 μg/ml (Table 1).

In an attempt to link the phenotypic resistance data with genotypic evidence of resistance, we searched for mutations known to confer antimicrobial resistance. Fig. 1 shows that for the two macrolides tested, azithromycin and erythromycin, there was concordance between MIC-based antimicrobial resistance profiles and the presence of SNPs in region V of 23S rRNA, nucleotides positions A2058 and A2059, known to confer resistance [33]. The 23S rRNA A2059G mutation and particularly the A2058G mutation also resulted in substantially increased MICs of the new fluoroketolide solithromycin, i.e., MIC 0.25–1 μg/ml and MIC 2 μg/ml, respectively. For the fluoroquinolones and correlating with ciprofloxacin and moxifloxacin resistance, the *parC* gene carried a characteristic fluoroquinolone resistance mutation in codon 83 that results in the amino acid substitution S83I (Fig. 1) [34]. However, although SNPs were found in *gyrAB* and *parE*, known to be associated with fluoroquinolone resistance in other Mycoplasmas [35], none of the SNPs were at known positions or correlated with the observed MIC-based resistance profiles.

All samples, including the reference, contained genes *dhfR* (MG_228) and *ksg*A (MG_463). The presence of these genes in other bacterial species confers resistance to trimethoprim [36] or kasgamycin [37] and chlarithromycin, [38], respectively. Although *dhfR* is often located on mobile genetic elements in other bacterial species, both of these genes appear to be part of the core genome in *M. genitalium* (and other Mycoplasmas), with no evidence of lateral gene transfer.

Four isolates (M2341, M6303, M6489 and M6711) showed slightly reduced susceptibility to doxycycline (MICs of 1–2 μg/ml; Table 1). We looked within the 11 ABC-family membrane transporter loci in the genome of *M. genitalium*, comprising more than 36 predicted CDSs [5, 39] to investigate the possibility of efflux related decrease susceptibility [40]. They are present in all our isolates, although there is some sequence variation (in this case, SNPs) that resulted in some CDSs being truncated (data not shown). Of the 4 samples showing reduced doxycycline susceptibility none possessed additional or unique ABC-transport systems or displayed any genetic variation that correlated to phenotypic resistance, in particular, no *tetM* gene homologs were identified.

## Discussion

This study shows the first phylogenetic framework for the species *Mycoplasma genitalium,* encompassing isolates from three continents which were collected over three decades (1980–2010). The phylogenetic tree subdivides the species into two main clades, of which Clade A includes the first genome sequenced strain G37^T^ [5]. Linking the isolate metadata to the phylogeny, there was no obvious correlation between phylogenetic position and either country of origin or the year of isolation, with both clades containing isolates from the three continents sampled (Europe, Australia and Asia (Japan)). However, whilst this is the largest collection of *M. genitalium* sequenced more intensive sequencing from more geographically and temporally diverse sources or collections would be needed to fully answer this question.

Comparing the genome assemblies and annotation of all isolates showed that genomes varied in size ~1 kb as an average (not excluding MgPar misassembled portions) with all isolates predicted to encode the same number of protein coding genes (*n* = 482), with no novel gene and/or other unique accessory regions with respect to the reference G37^T^ genome [5, 27]. Likewise, there was no evidence of the presence of extra chromosomal elements in any of the genomes sequenced. Five of the strains sequenced, the ATCC: R32G, TW10-6G, TW10-5G, TW48–5G [25] and UTMB-10G [26], presented negligible differences between them and to the reference G37^T^ genome. It is important that the *M. genitalium* research community becomes aware of this fact. Although the clinical samples from which they were isolated were collected in 1974–5 [25] and 1986 [26], their isolation took place in 1987 in the same laboratory where G37^T^ was potentially used at the same time as a positive control for the growth conditions and other reference strains may have been cross-contaminated with G37^T^. This is certainly consistent with the sequence data, and the reason our subsequent analysis was carried out without these five sequences.

Using SNP density as a predictor of recombination it is evident that recombination appears non-uniform and as hotspots distributed across our genomes, a fact that has been shown previously [28]. Furthermore, the different sub-lineages found show different patterns of recombination. For example, Clade B carries a unique recombination region stretching from *gyrA* to MG_009 (encompassing essential genes encoding for DNA topology, and DNA and tRNA synthesis proteins), and different regions have been subjected to different numbers of recombination events when compared across all isolates. It is clear that recombination is not restricted to MgPar loci, with other cytoadherence and lipoprotein proteins being included [5, 30], as well as housekeeping genes involved in DNA topology, like the region mentioned (region I) shared by clade II isolates, which includes the GyrA function.

The nine MgPar regions show the highest level of recombination, consistent with previous findings [5], with the three genomic regions showing the highest number of recombination events represented by the block composed of MgPar4, *mgpABC* and MgPar5, the block composed of MgPar2 and MgPar3, and the regions adjacent and containing MgPar1. Through this recombination, which we have shown as both reciprocal and non-reciprocal intramolecular events, in accordance with previous findings [15, 18, 29, 31], the MgPar regions represent a reservoir of variation that can be rapidly introduced into the expression site genes *mgpBC,* and through recombination between MgPar loci create further diversity in the MgPar loci themselves. The resulting *mgpBC* variability translates into antigenic variation that permits *M. genitalium* to evade the host immune system in order to establish the infection and its persistence [18].

Unlike the MgPar regions, the other recombinogenic regions illustrated along the genome are not repeated and accordingly, they cannot follow the same model. Looking at all recombinogenic regions, including *mgpBC* and the MgPar loci, based on our default mapping cut-off of >91%, all these regions share more sequence identity with orthologonal loci in *M. genitalium* isolate genomes than to any other *Mycoplasma* species*.* However, this may not be too surprising since it is an extremely diverse genus with orthologues in *M. pneumoniae* being the most similar sharing on average 69% nucleotide identity (our own data) or 75.9% protein similarity [4]. Therefore, these recombined sequences within the MgPar/*mgpBC* and elsewhere must emanate either from an unknown close relative of *M. genitalium* or from the wider diversity of the species – still not captured in the strains we currently have sequenced.

Searching for genotypic evidence of antimicrobial resistance with no evidence of the acquisition of genes associated with AMR, we looked for mutations known to confer antimicrobial resistance. These data confirmed the presence of SNPs at nucleotide positions A2058 and A2059 in region V of the 23S rRNA and was in concordance with the phenotypic MIC data for the two macrolides tested, azithromycin and erythromycin and to other reports [33]. Similarly, for the fluoroquinolones, we confirmed a SNP in the codon 83 for the *parC* gene that would result in the S83I amino acid substitution, correlating with ciprofloxacin and moxifloxacin resistance [34] but no SNPs were found in *gyrAB/parE* specific reported positions for Mycoplasmas [35]. These data also showed a complete absence of acquired resistance genes in these isolates, which genes such as *dhfR* and *ksgA*, conferring various resistances in other bacteria [36–38], appearing to be part of the core genome of *Mycoplasma genitalium.* No genetic factors have been shown to confer doxycycline resistance in *M. genitalium,* although for *M. hominis* it has been previously suggested that genes encoding membrane transport functions such as ABC-family membrane transport proteins could be involved in conferring resistance to these antibiotics through efflux [40]. However, none of the four strains showing reduced susceptibility to this antibiotic displayed genetic variations correlating with it.

Although we have limited power to understand the impact of antimicrobial resistance on the population structure of *M. genitalium* in this study, when the phenotypic and genotypic resistance profiles were added to the whole genome phylogeny, it was clear that, antimicrobial resistance was not associated to a specific clade in the tree and has emerged independently on multiple occasions.

## Conclusion

In this present work, we have increased the number of distinct genomes for the fastidious and slow-growing *M. genitalium* from 5 to 26. All isolates were cultured and were with known provenance. This provides a much-needed snapshot of the population framework of *M. genitalium* collected between 1980 and 2010 and over multiple continents; it underlines the huge degree with which recombination has and is shaping the evolution of this species and not just in the MgPar loci. Avoiding the hotspots of recombination is also important when designing molecular tests for diagnosis and typing of *M. genitalium,* at a time when antibiotic resistance increases both in clinical and non-clinical settings.

## Methods

### *M. genitalium* strains, culture history and DNA extractions

A total of 22 *M. genitalium* isolates were obtained from 20 patients attending clinics in Europe, Asia, and Australia (Table 1) by primary inoculation into Vero cell cultures and monitoring growth by qPCR as previously described [23, 24, 41]. After a varying number of passages in the Vero cell culture system, *M. genitalium* was sufficiently adapted to establish growth in cell free mycoplasma medium. The isolates were single colony cloned three times to ensure purity. Three strains were isolated from the same French patient; their clinical samples were kindly provided by Dr. Bertille de Barbeyrac, Bordeaux, France. The M30 early strain was obtained in its 7th passage from The Mollicutes Collection of Cultures and Antisera, Gainesville, as a freeze-dried culture. This early passage of one of the two original isolates [1] is clearly distinct from the M30 strain available from the ATCC [41]. Five isolates available from the ATCC described as extra-genital (R32G, TW10-6G, TW10-5G, TW48–5G [25] and UTMB-10G [26] were obtained directly from Dr. Joseph Tully who also deposited the strains in the ATCC.

All strains were grown at 37 °C in 150 ml of modified Friis’ FB medium [24] in 800-ml Nunclon™ disposable polystyrene culture flasks (ThermoFisher Scientific, Waltham, MA, USA). Adherent mycoplasmas from four flasks were harvested by centrifugation in the late log phase. DNA was extracted from the cell pellets using the QIAamp® DNA Mini Kit (Qiagen, Hilden, Germany) according to the manufacturer’s instructions. DNA concentrations were measured with the Qubit dsDNA HS Assay Kit (ThermoFisher Scientific).

### Genome sequencing and de novo assemblies

DNA libraries for all samples were prepared according to published protocols [42, 43] and sequenced on an Illumina MiSeq operated platform according to the manufacturer’s instructions at the Wellcome Trust Sanger Institute. Total bases of raw data per sample are recorded in Additional file 1: Table S1. An average of 135.02 Mb of sequence data was obtained per sample in 150 bp read pairs. De novo genome assemblies were obtained from Illumina fastq files using an in-house implemented pipeline with detailed stages previously published [44] and can be downloaded from our ftp site [45]. Artemis [46] and ACT (Artemis Comparative Tool, [47]) were used as genome browsers for manual checking and comparison of genome assemblies.

### Mapping, single nucleotide polymorphism (SNP) calling and phylogenetic tools

Phylogenetic analysis was performed using the isolates read data (this study) and the published [22] genomes of samples M2321, M6280, M6320 and M2288 (accession numbers [GenBank: CP003770–3], respectively). Fastq data was generated from published genomes by simulating short-read data in silico*.* Read mapping was performed using SMALT [48] against the sequence of *M. genitalium* strain G37^T^ (accession number [GenBank: L43967]) using a lower cut off for mapping of ~91% nucleotide identity [5, 49], ignoring exact repeats; for mapping parameters see Additional file 1: Table S1). Single nucleotide polymorphisms (SNPs) were identified using an in-house script as previously described [50]. Gubbins [51] was run on the whole genome alignment in order to identify regions of recombination (defined by SNP density) which were masked out so as to produce a recombination-free phylogeny [50, 52]. The impact of recombination was also assessed using the maximum χ^2^ test, neighbour similarity score (NSS) and the pairwise homoplasy index (PHI) using the PhiPack package and the *M. genitalium* whole genome alignment [53]. Phylogenetic trees were constructed using RAxML assuming a general time reversible site model with gamma correction [54]. Support for clades in the maximum likelihood tree was assessed by running the same analysis on 100 bootstrap replicates.

*M. pneumoniae* strain M129 (accession number U00089), a member of the most closely related *Mycoplasma* species [27, 55] was used to define the root position for the *M. genitalium* phylogenies by constructing a phylogeny based on core gene SNPs from a *M. pneumoniae-M. genitalium* pan-genome using Roary (v3.7.0) [56] with a minimum protein identity of 75% and genes aligned using MAFFT (v7.205) [57]. This resulted in a predicted core genome of 264 genes/ortholog groups equating to a core of 174,384 bases which is 30% of the size of *M. genitalium* genome. The corresponding alignment can be downloaded as a DOI file [58].

SEAVIEW (v.4.2), [59] was used for sequence alignments and visualization. Phylogenetic groups were defined using Bayesian Analysis of Population Structure, performed on the variant sites alignment using the BAPS individual mixture model [60].

*Tanglegrams* comparing tree topologies were prepared using Dendroscope (v.3.2.10) [61].

SEAVIEW, using MUSCLE, was used to align the sequences of the MgPar or *mgpBC* loci, which were taken from each genome assembly. RAxML was used to construct bipartition trees from these alignments using parsimony, the final un-rooted phylogenetic tree was used to determine SNPs per branch [44]. Where assembled sequences of non-typical MgPar regions were not certain due to high levels of recombination, they were excluded from the analysis.

### Antimicrobial resistance testing

The minimum inhibitory concentrations (MICs) of azithromycin, erythromycin, moxifloxacin, ciprofloxacin, doxycycline, and solithromycin were determined using a Vero cell culture based method as previously described [62, 63]. SNPs associated with resistance were investigated by extracting the sequences of the relevant rRNA or housekeeping gene from each sample’s assembly by using an *in-silico* PCR script.

## Additional files

Additional file 1: Table S1. Illumina sequenced genomes of *M. genitalium* isolates and related genomes: mapping and assembly statistics and genome characteristics. (XLSX 20 kb)

Additional file 2: Table S2. Recombination regions identified and removed for the *Mycoplasma genitalium* phylogeny. (XLS 89 kb)

Additional file 3: Figure S1. Dendogram output bipartitions tangled trees representing changes on topology before (left) and after (right) running Gubbins. (PDF 124 kb)

Additional file 4: Figure S2. Bootstrapping values for the *M. genitalum* phylogenetic tree represented in Fig. 1. (PDF 852 kb)

Additional file 5: Figure S3. Path-O-Gen output plots. The plots are showing the root to rip distances vs. year of isolation of all *Mycoplasma genitalium* strains (left) and the frequency distribution after a 100 permutations (right). (PDF 6 kb)

Additional file 6: Figure S4. Sequence homology representation of the *mgpB* and *mgpC* genes repeats positions and their homologous sequences in MgPar regions. Homologue repeat positions in each of the different structured MgPar region are highlighted in the same colours. All based on the M. genitalium G37^T^ genome. Dotted vertical lines represent restriction fragments and hatched boxes represents intervening sequences that are unusually A-T rich and contain stop codons [15]. (PDF 114 kb)

Additional file 7: Figure S5. RAxML Phylogenetic trees reconstructed with parsimony for the five homologous repeat sequences contained in the *mgp* operon and the MgPar regions for isolates of the same patient. Sequences were coloured when they were not the same in the three isolates. Numbers over the scale line represent SNPs. For the EF sequences, as an example, all SNPs with respect to the G37^T^ EF sequence at the *mgp* operon location are plotted after reconstruction on the right. As identical samples contain identical SNPs profiles, it is easy to spot blocks of sequence replacements due to recombination. One reciprocal recombination is marked with a cross for the two locations where it happened within the same sample. Other blocks do not have reciprocal counterparts, a sign of multiple recombination steps or a unique non-reciprocal recombination event. (PDF 195 kb)

Additional file 8:Figure S6. RAxML Phylogenetic trees reconstructed with parsimony for the five homologous sequences contained in the *mgp* operon and the MgPar regions of *Mycoplasma genitalium* genomes. Numbers over the scale line represent SNPs. Clustering of sequencing by sample or genomic location has been emphasized by displacing rectangles within the metadata columns. (PDF 539 kb)

## Abbreviations

%GC: Percentage of Guanine and Cytosine content
ACT: Artemis Comparative Tool
ATCC: American Type Culture Collection
AZM: Azithromycin
CDS: Coding Sequence
CIP: Ciprofloxacin
DOX: Doxycycline
EMBL: European Molecular Biology Laboratory
ERY: Erythromycin
MIC: Minimum Inhibitory Concentration
MXF: Moxifloxacin
NAATs: Nucleic Acid Amplifications Tests
NGU: Non-gonococcal Urethritis
rRNA: ribosomal RiboNucleic Acid
SNP: Single Nucleotide Polymorphism
SOL: Solithromycin
