# Supplementary figures and images for: Mycoplasma genitalium: whole genome sequence analysis, recombination and population structure
Source: BMC Genomics. 2017 Dec 28;18:993. doi: 10.1186/s12864-017-4399-6 (PMC5745988; doi:10.1186/s12864-017-4399-6)

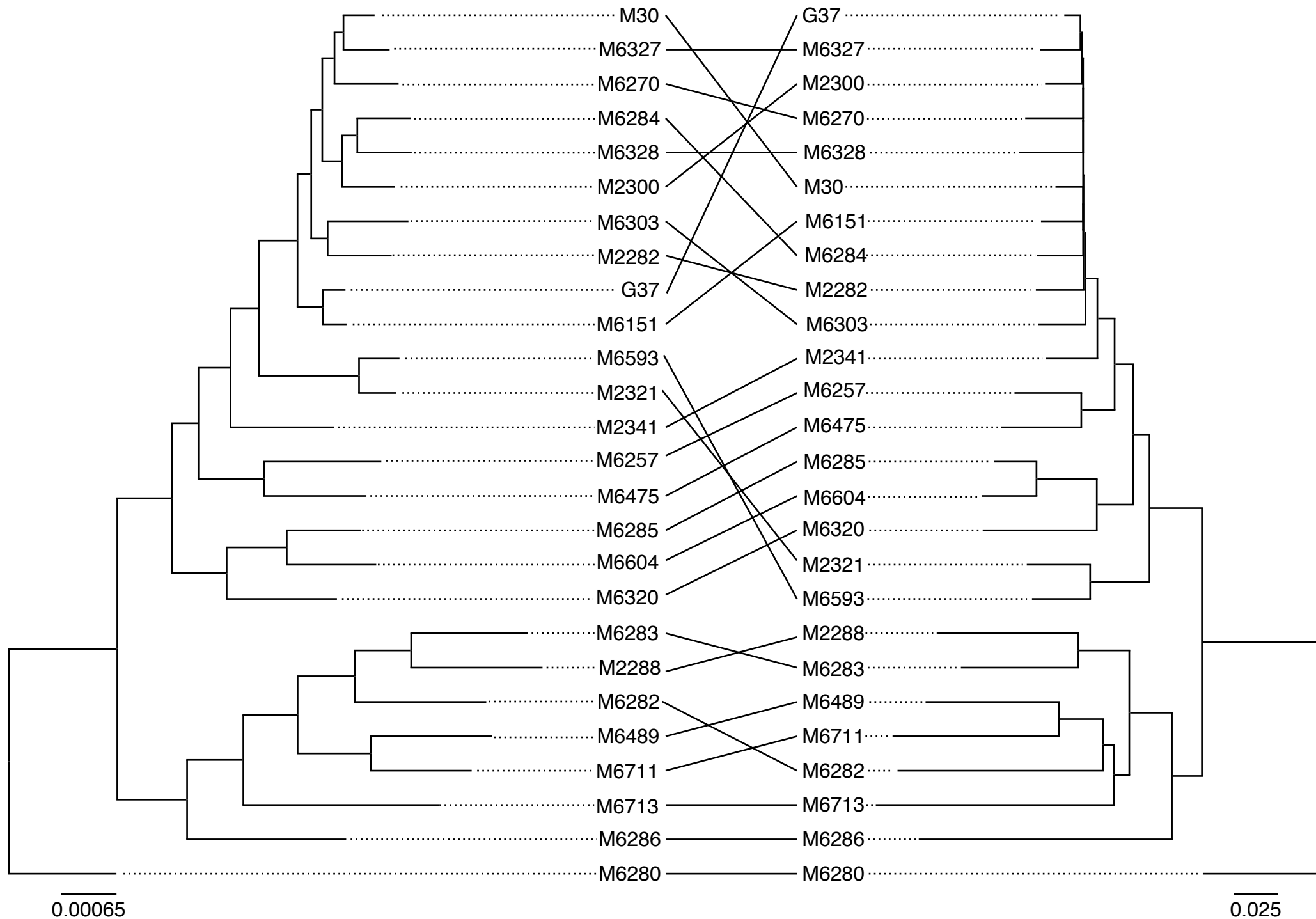

Supplement: Supplementary file 3 — Dendogram output bipartitions tangled trees representing changes on topology before (left) and after (right) running Gubbins. (PDF 124 kb) [file 12864_2017_4399_MOESM3_ESM.pdf]

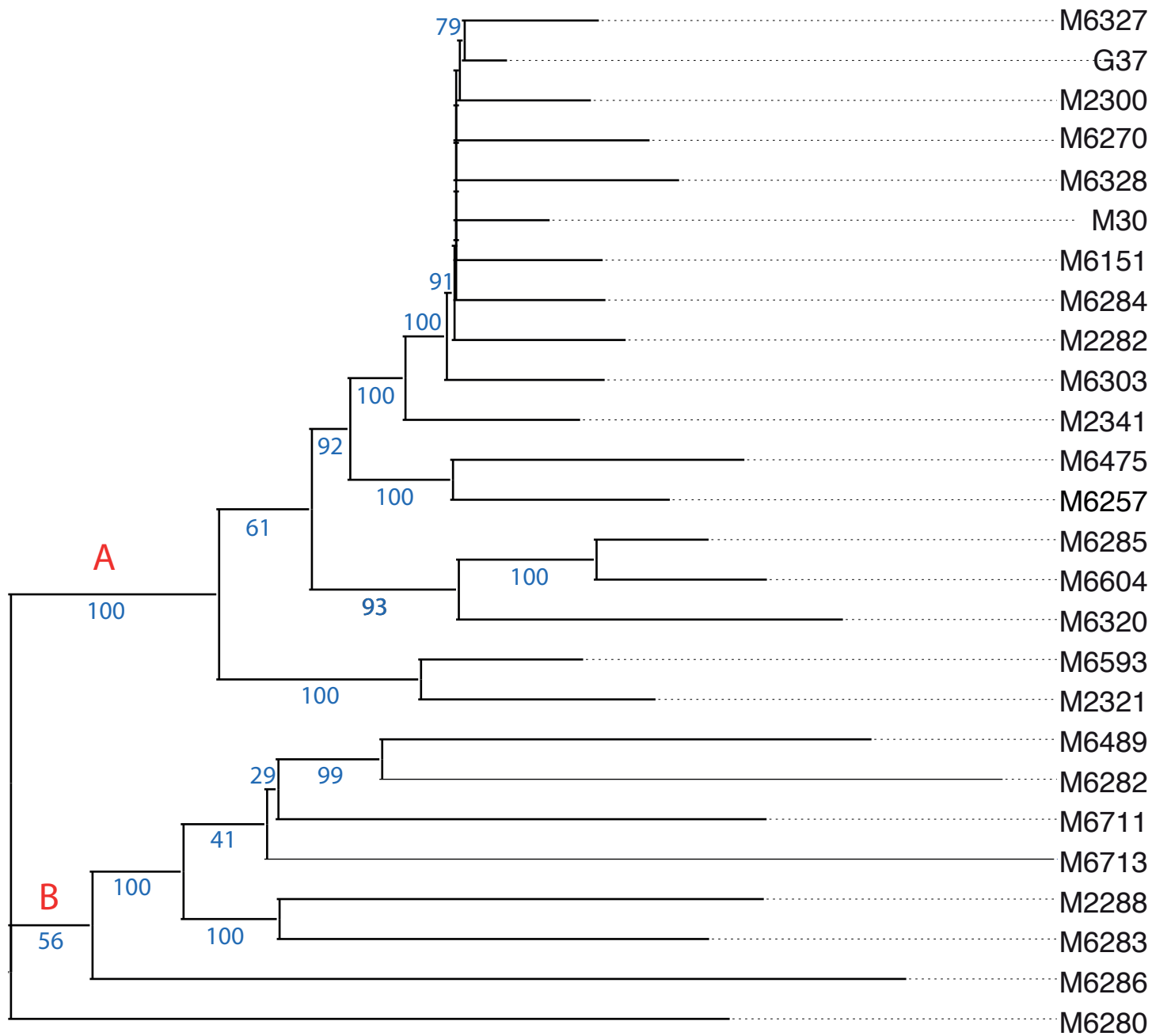

Supplement: Supplementary file 4 — Bootstrapping values for the M. genitalum phylogenetic tree represented in Fig. 1. (PDF 852 kb) [file 12864_2017_4399_MOESM4_ESM.pdf]

# Path-O-Gen Regression

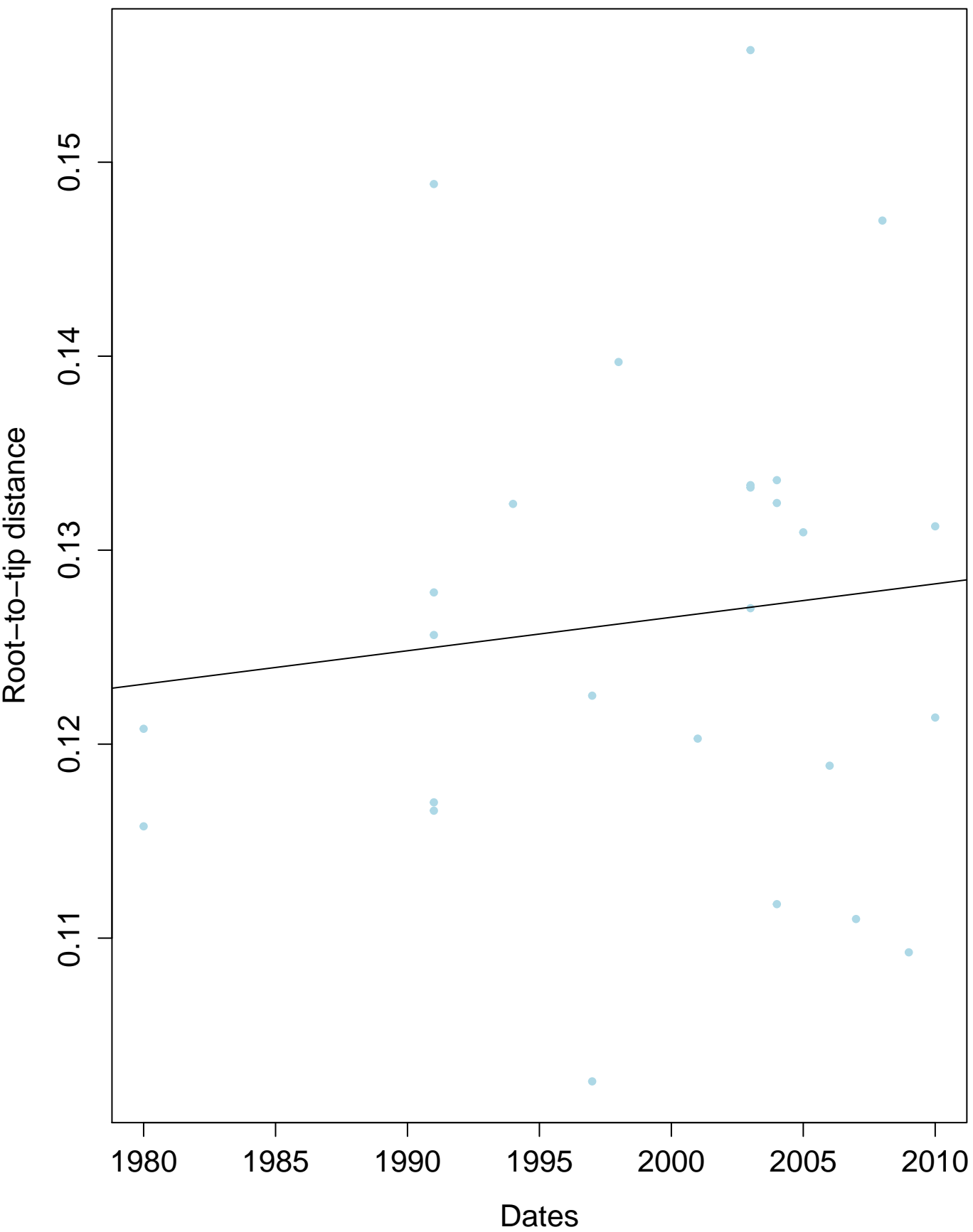

# Randomised data sets

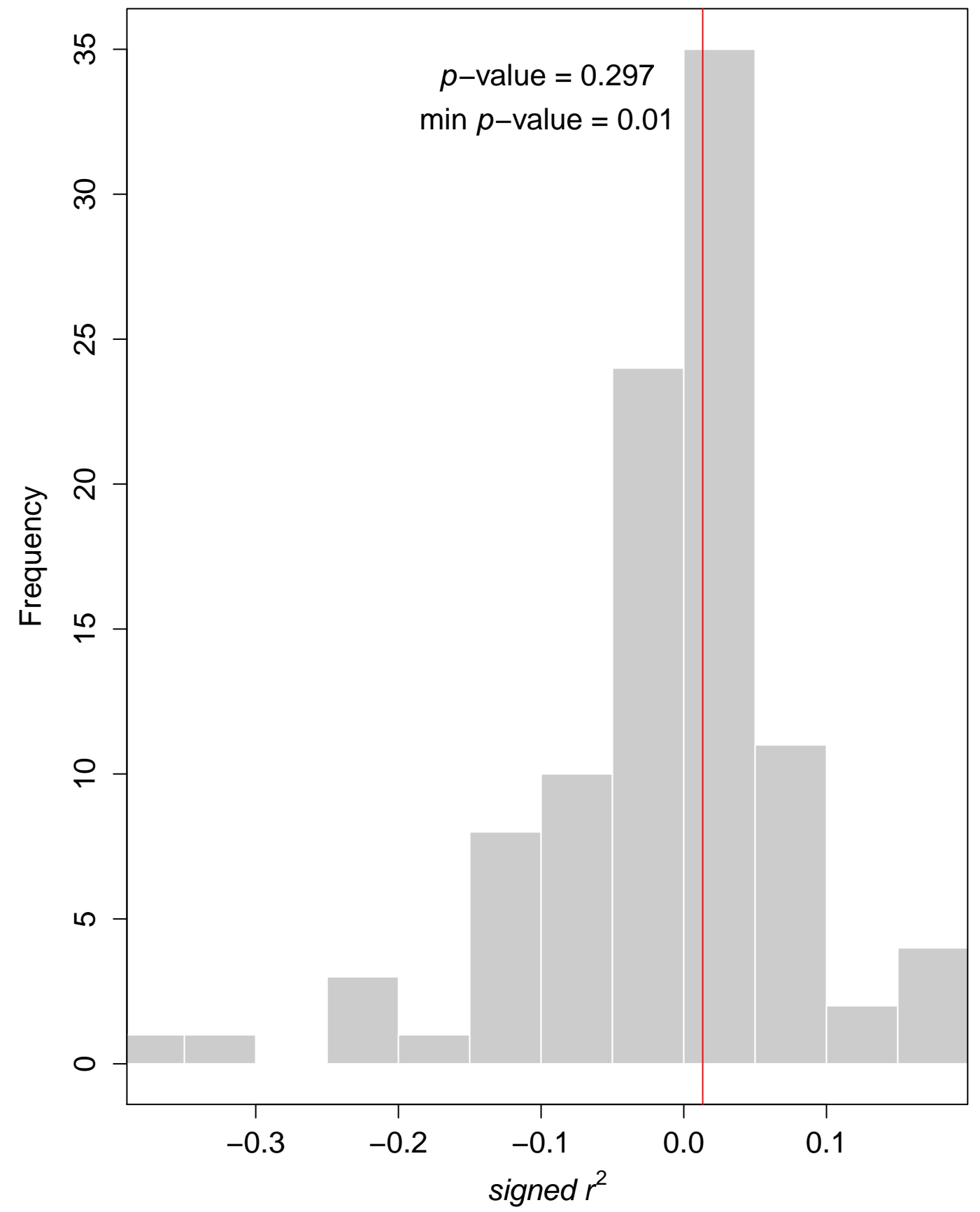

Supplement: Supplementary file 5 — Path-O-Gen output plots. The plots are showing the root to rip distances vs. year of isolation of all Mycoplasma genitalium strains (left) and the frequency distribution after a 100 permutations (right). (PDF 6 kb) [file 12864_2017_4399_MOESM5_ESM.pdf]

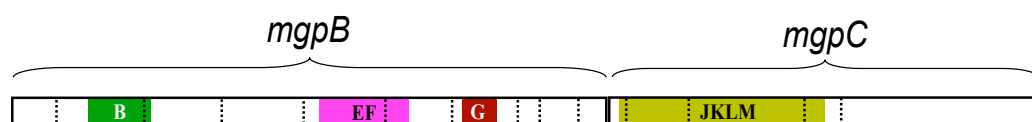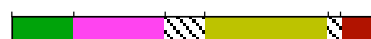

MgPar1

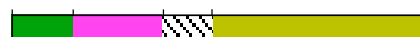

MgPar2 (MgPar8)

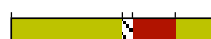

MgPar3

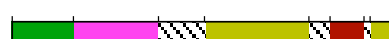

MgPar4 (MgPar5, MgPar7)

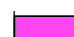

MgPar6

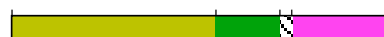

MgPar9

Supplement: Supplementary file 6 — Sequence homology representation of the mgpB and mgpC genes repeats positions and their homologous sequences in MgPar regions. Homologue repeat positions in each of the different structured MgPar region are highlighted in the same colours. All based on the M. genitalium G37T genome. Dotted vertical lines represent restriction fragments and hatched boxes represents intervening sequences that are unusually A-T rich and contain stop codons [15]. (PDF 114 kb) [file 12864_2017_4399_MOESM6_ESM.pdf]

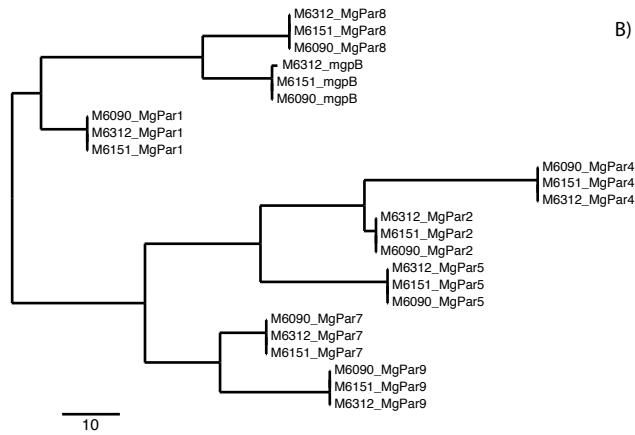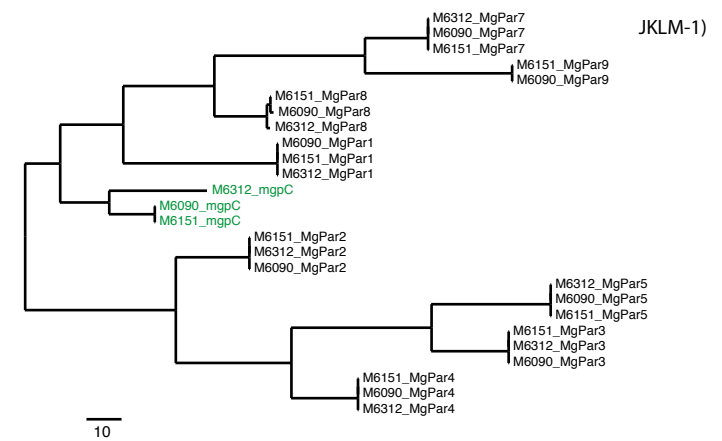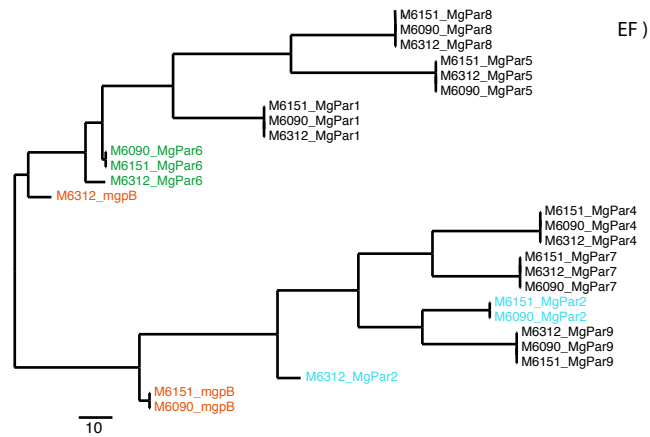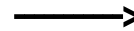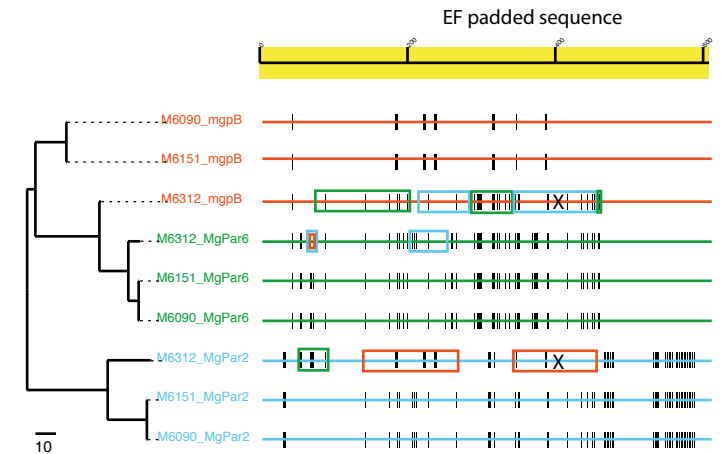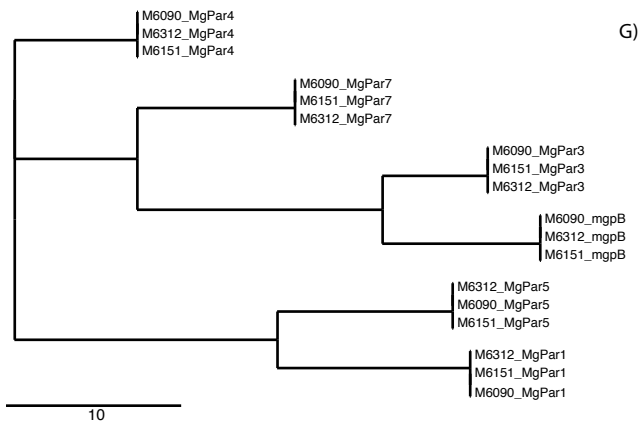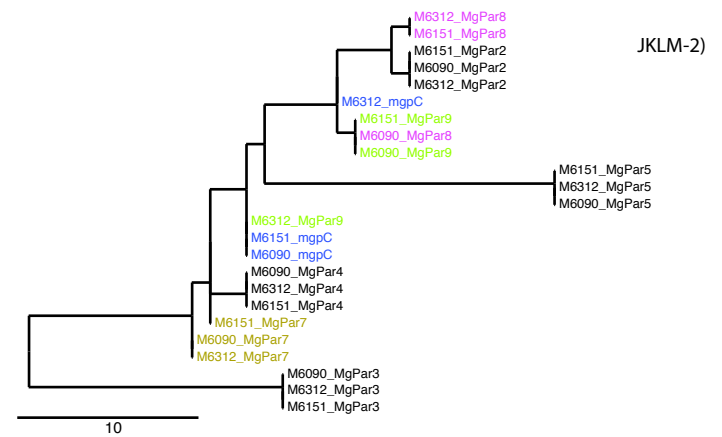

Supplement: Supplementary file 7 — RAxML Phylogenetic trees reconstructed with parsimony for the five homologous repeat sequences contained in the mgp operon and the MgPar regions for isolates of the same patient. Sequences were coloured when they were not the same in the three isolates. Numbers over the scale line represent SNPs. For the EF sequences, as an example, all SNPs with respect to the G37T EF sequence at the mgp operon location are plotted after reconstruction on the right. As identical samples contain identical SNPs profiles, it is easy to spot blocks of sequence replacements due to recombination. One reciprocal recombination is marked with a cross for the two locations where it happened within the same sample. Other blocks do not have reciprocal counterparts, a sign of multiple recombination steps or a unique non-reciprocal recombination event. (PDF 195 kb) [file 12864_2017_4399_MOESM7_ESM.pdf]

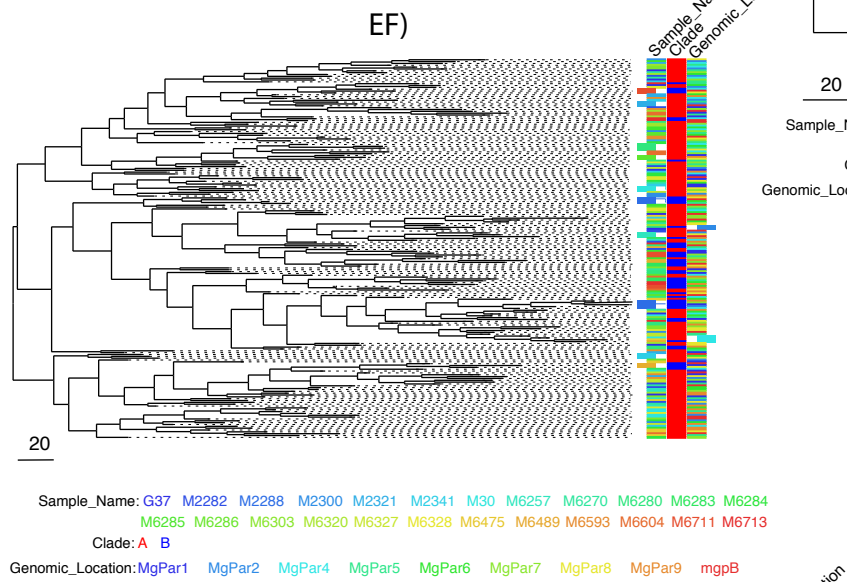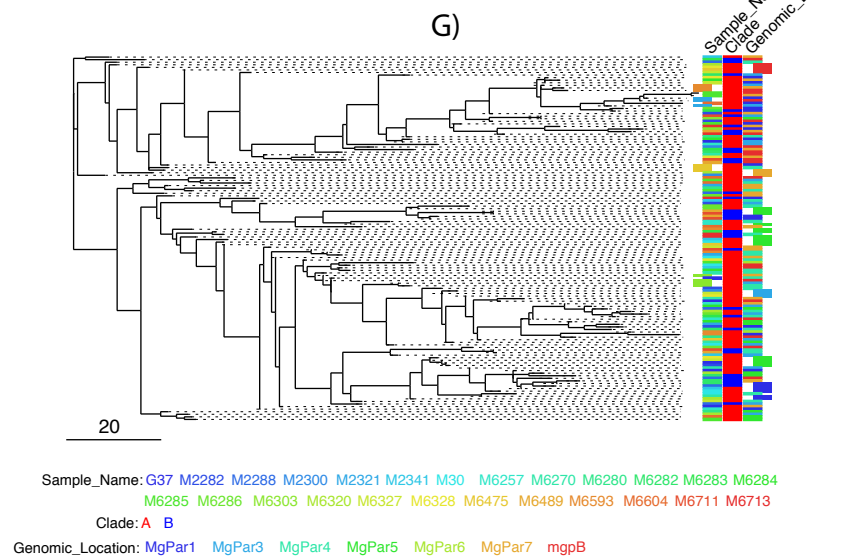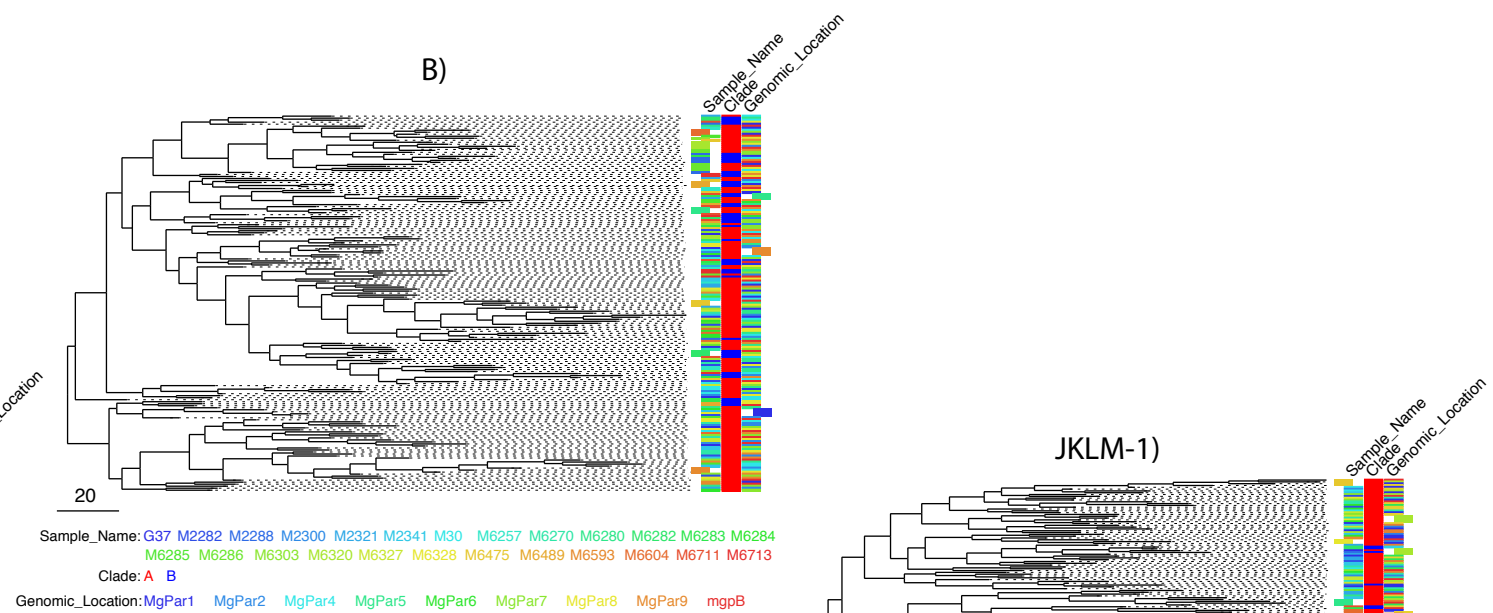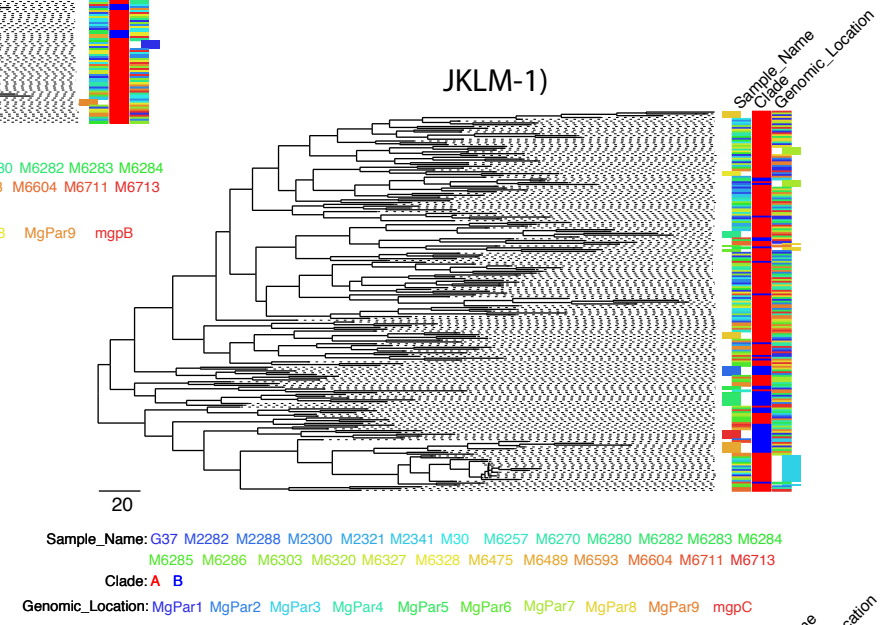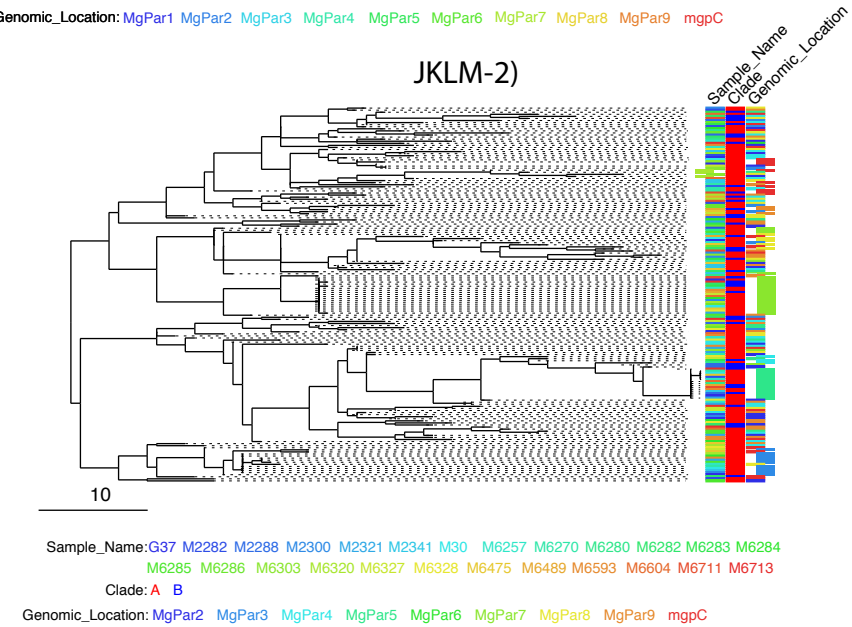

Supplement: Supplementary file 8 — RAxML Phylogenetic trees reconstructed with parsimony for the five homologous sequences contained in the mgp operon and the MgPar regions of Mycoplasma genitalium genomes. Numbers over the scale line represent SNPs. Clustering of sequencing by sample or genomic location has been emphasized by displacing rectangles within the metadata columns. (PDF 539 kb) [file 12864_2017_4399_MOESM8_ESM.pdf]
